# A huge and invasive skull metastasis caused by renal cell carcinoma

**Published:** 2017-04-04

**Authors:** Ali Babashahi, Morteza Taheri

**Affiliations:** Department of Neurosurgery, Rasool Akram Hospital, Iran University of Medical Sciences, Tehran, Iran

**Keywords:** Skull, Metastasis, Renal Cell Carcinoma, Neoplasms, Magnetic Resonance Imaging

A 65-year-old man presented by a progressive scalp mass from 9 months before. Physical examination showed a huge mass on his skull (Figure 1) associated with two small swellings in his axilla and flank. 

**Figure 1:**
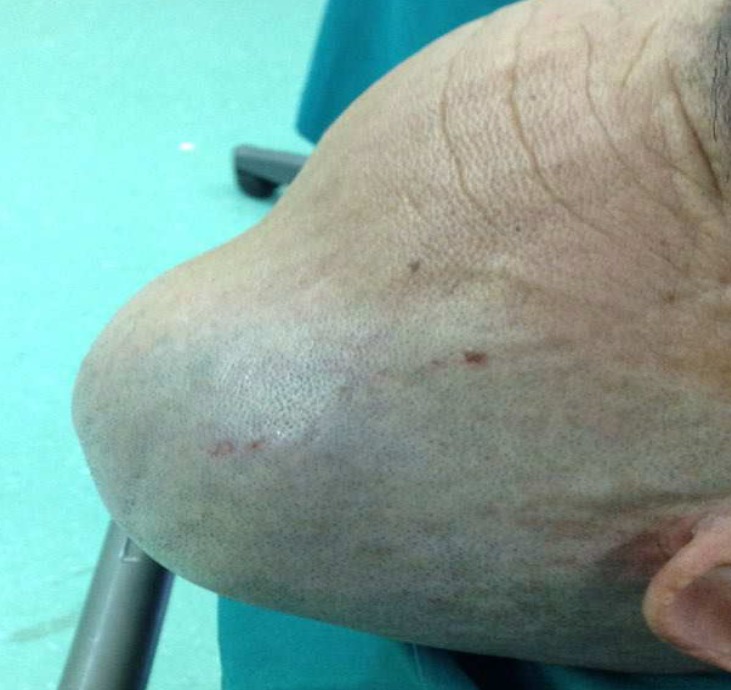
The image of the patient's scalp

Neurological examination was normal. The laboratory data and preoperative evaluation was normal, too. The brain computed tomography (CT-scan) and magnetic resonance imaging (MRI) demonstrated a huge extra-axial mass in right parietal bone resulted to bone destruction and scalp invasion (Figures 2 and 3). 

**Figure 2:**
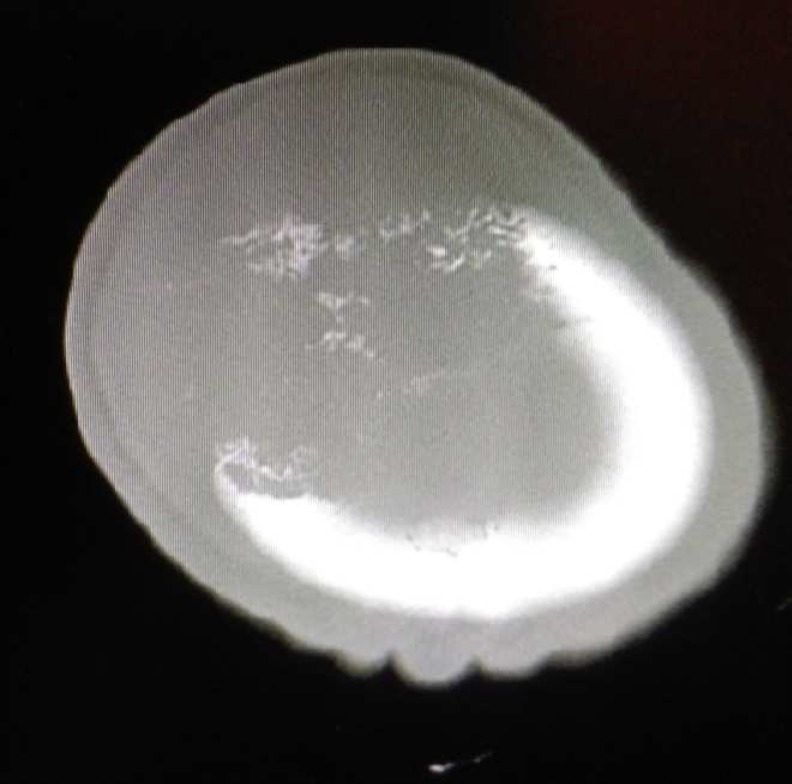
The bone window brain computed tomography (CT-scan)

In surgery, there was a huge oval-shape hemorrhagic and firm mass associated with scalp invasion and bone destruction (Figure 4). 

**Figure 3:**
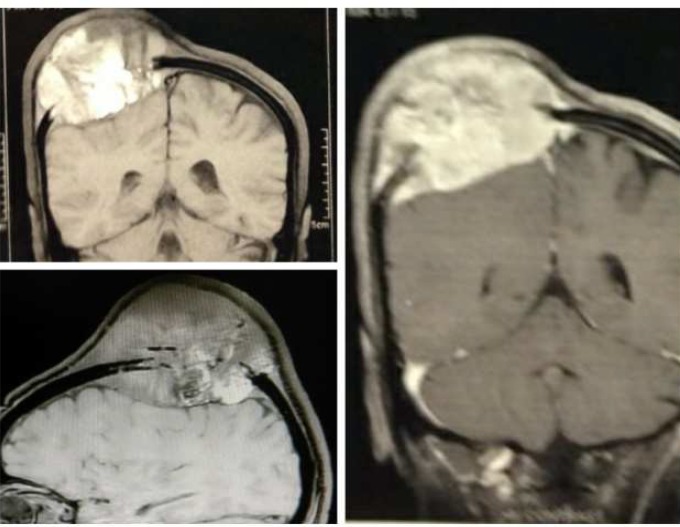
The coronal T1, sagittal FLAIR, and coronal Gd-enhancement T1 image sequences of brain magnetic resonance imaging (MRI)

After skin incision, the mass was dissected from scalp and debulked; parietal craniectomy around the lesion was done; dura was opened and tumor was dissected from neural tissue and was resected totally. Helping synthetic patch and titanium mesh, the dura and skull were repaired. Histopathology revealed renal cell carcinoma (RCC).

**Figure 4:**
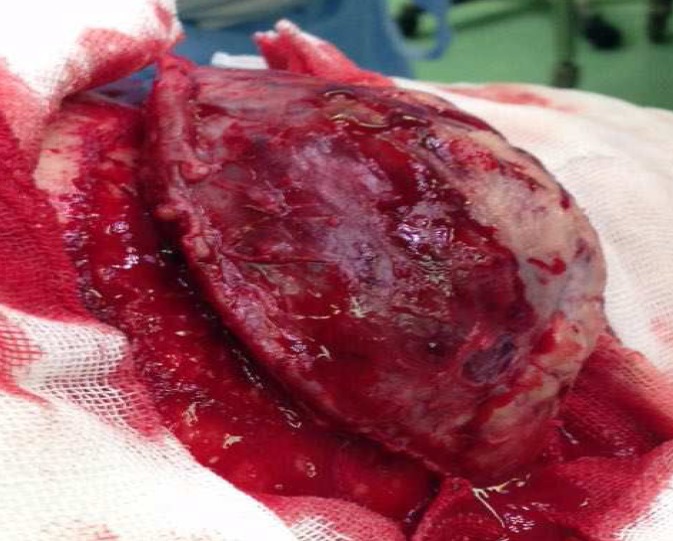
Intraoperative photograghy of the lesion

Sepulveda, et al. reported a 62-year-old man presented with a large expansive and destructive mass within cranial base associated with bilateral internal carotid artery involvement. Incisional biopsy revealed clear cell adenocarcinoma in favor of renal tumor metastasis.^1^

Rekhi, et al. reported a 15-year-old girl presented with a painless swelling in the occipital region. She had a history of excisional biopsy with a diagnosis of an alveolar soft part sarcoma. The lesion recurred within one month. CT-scan revealed a vascular destructive skull mass compressed the cerebellum. Metastatic RCC was ascertained after excisional biopsy and systemic work up.^2^

Yeh, et al. presented an 80-year-old man admitted for an incidental mass on the left parietal. The CT-scan revealed a destructive soft tissue lesion in the left parietal with intracranial and scalp invasion. Needle biopsy demonstrated metastatic RCC.^3^

Koutnouyan, et al. reported a 33-year-old woman who was referred for a mass in forehead region as a benign lesion. Because of intraoperative severe bleeding and presence of bone erosion, only biopsy rather than resection was done. Histopathology and metastatic work-up demonstrated metastatic RCC.^4^

A metastatic lesion should be in differential diagnosis of a destructive skull lesion with brain and scalp invasion, although it occurs rarely.

## Notes:

**How to cite this article:** Babashahi A, Taheri M. A huge and invasive skull metastasis caused by renal cell carcinoma. Iran J Neurol 2017; 16(2): 103-4.
